# The Influence of Anionic Initiator on the Selected Properties of Poly-*N*-Isopropyl Acrylamide Evaluated for Controlled Drug Delivery

**DOI:** 10.3390/molecules22010023

**Published:** 2016-12-26

**Authors:** Agnieszka Gola, Tomasz Knysak, Witold Musial

**Affiliations:** Department of Physical Chemistry, Pharmaceutical Faculty, Wroclaw Medical University, Borowska 211, 50-556 Wroclaw, Poland; agnieszka.gola@umed.wroc.pl (A.G.); tomekknysak06@interia.eu (T.K.)

**Keywords:** nanospheres, *N*-isopropyl acrylamide, volume phase transition temperature, electrical conductivity

## Abstract

The aim of the study was to monitor the influence of increasing initiator concentrations on the properties of poly-*N*-isopropylacrylamide (polyNIPA) nanoparticles obtained via surfactant free precipitation polymerization (SFPP). In all studied systems P-001 to P-1, the same amount of monomer was used, and increasing amounts of potassium persulphate (KPS). The course of each reaction was monitored by measuring the conductivity of the whole system. The resulting composition of products was confirmed by attenuated total reflectance within Fourier transformed infrared spectroscopy (ATR-FTIR) measurements. The hydrodynamic diameters with polydispersity index (PDI) and zeta potential (ZP) were measured in aqueous dispersions of the synthesized polymers in dynamic light scattering (DLS) device (λ = 678 nm), and were found to be for P-1: 20.33 nm (PDI = 0.49) and −7 mV, for P-05: 22.24 nm (PDI = 0.39) and −5 mV, for P-01: 50.14 nm (PDI = 0.49) and −3 mV, for P-005: 62.75 nm (PDI = 0.54) and −3 mV and for P-001: 509.4 nm (PDI = 0.61) and −12 mV at 18 °C, respectively. Initiator concentration affects the size and ZP of particles. The hydrodynamic diameter decreases with initiator concentration increase, whereas the time of the reaction decreases when the initiator concentration increases. This fact is reflected in the observed values of conductivity in the course of the performed reaction. Evaluated volume phase transition temperature in the range of 32 °C enables further research of the nanoparticles as thermosensitive drug carriers.

## 1. Introduction

The potential application of *N*-isopropylacrylamide (NIPA) derivatives in controlled release of active pharmaceutical ingredients (API) is presently intensively studied [1]. Some interesting potential applications include the controlled release of: doxorubicin [2], mesenchymal stem cells [3], basic fibroblast growth factor [4], theophylline [5], epirubicin [6], prodigosin [7], and simvastatin [8]. The NIPA derivatives may act as “smart” polymers, sensitive to a temperature factor, releasing the API, when the temperature increases locally [9,10,11]. During the surfactant free precipitation polymerization (SFPP) of NIPA derivatives, the radical polymerization is initiated by free radicals, and consequently the oligomer chain grows. In the course of the particle growth, the chains obtain—after a specified time—high molecular mass, at which the turbidity is usually observed [12]. The initial initiator level has a crucial influence on the number and molecular mass of synthesized nanoparticles. The application of appropriate initiation conditions enables control of the properties of obtained macromolecules [13,14]. Some authors directly investigated the influence of cationic initiator on the results of the synthesis of polymeric derivatives of NIPA, including the number and the size of obtained particles [15,16]. The functional groups implemented into NIPA polymer as co-monomers affect the possibilities of drug binding to the polymer. The carboxyl groups may enable ionic binding of cationic drugs [5,17], whereas addition of anionic co-monomer should consequently support binding of acidic APIs. The NIPA derivatives undergo volume phase transition at temperature, which is usually close to the range of human physiological temperatures [7,18,19,20]. Most of the known volume phase transition temperatures (VPTT) for NIPA derivatives are around 32 °C. The phenomenon of phase transition enables drug release followed by a temperature trigger. The synthesis of NIPA derivatives is a radical polymerization, with a characteristic polymerization period, which usually consumes around 7–10 h in the case of the synthesis of a polymer without any additional co-monomers. The monitoring of the process is important, as the yield of the SFPP depends on time. The knowledge on approximate time of full saturation of the vinyl bonds present in the system would be advantageous for the proper course of the synthesis. The practical recognition of the final stage of the reaction would also diminish the time of the purification of the resulting polymer. Some approaches to resolve this problem were proposed in the last decade, however there is still a lack of wide spectrum of data for NIPA derivatives synthesis. Some authors directed their interest into monitoring of some polymerization processes via on-line conductivity monitoring, however the data obtained with styrene polymerization, for example, were not very promising. In this study, we aimed to monitor the synthesis of the potential nanostructured thermosensitive drug carriers prepared with the use of anionic initiator via conductivity measurements. We also aimed to compare the results of conductivity measurements with selected properties of obtained polymeric particles.

## 2. Results

### 2.1. Synthesis Course

In the experimental design, five samples of NIPA derivatives were synthesized—P-1, P-05, P-01, P-005, and P-001—with decreasing concentrations of anionic initiator, potassium persulphate (KPS), respectively: 2.2 × 10^−2^, 1.1 × 10^−2^, 2.2 × 10^−3^, 1.1 × 10^−3^, 2.3 × 10^−4^ M·dm^−3^. The same conditions of temperature and reaction environment were applied for evaluated samples. In all samples, after several minutes of initiation, a specific turbidity was observed which persisted during the SFPP course, and retreated after cooling of the samples down to room temperature.

### 2.2. Fourier Transform Infrared Spectroscopy Measurements

**Figure 1:**
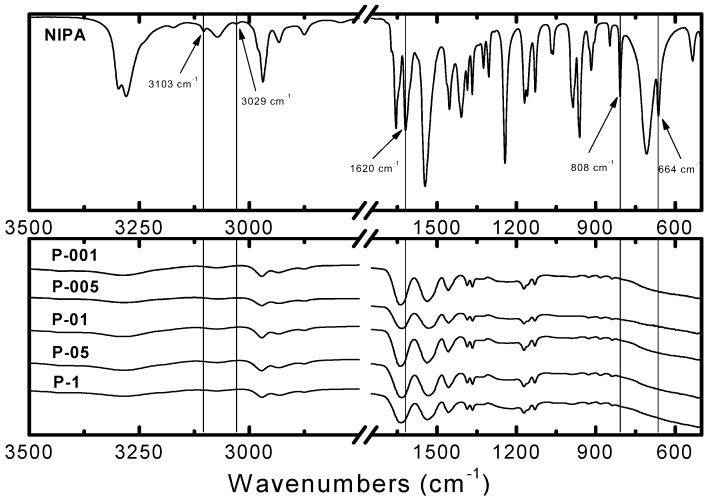
Fourier transformed infrared spectroscopy with attenuated total reflectance (ATR-FTIR) spectra of monomer *N*-isopropylacrylamide (NIPA) (**Top**); and five synthesized polymers with decreasing concentration of initiator: P-1, P-05, P-01, P-005, P-001 (**Bottom**).

The Fourier Transform Infrared Spectroscopy Measurements (FTIR) spectra of five NIPA polymers, prepared with increasing contents of initiator, compared to the spectrum of NIPA monomer, were depicted in Figure 1.

Characteristic absorption bands of unsaturated groups of C=C in FTIR spectrum of NIPA were observed at 3103, 3029 cm^−1^ [21]. Parallely at 1620 cm^−1^, 808 cm^−1^, and at 664 cm^−1^, were observed signals of stretching vibrations H–C=C [22], H–C=C out-of-plane deformation vibration bond conjugated to the C=O, and wagging vibrations H–C=C [23], respectively. The same bands were not observed in the spectra of P-1, P-05, P-01, P-005, and P-001 polymers.

### 2.3. Nuclear Magnetic Resonance Spectroscopy Measurements

**Figure 2:**
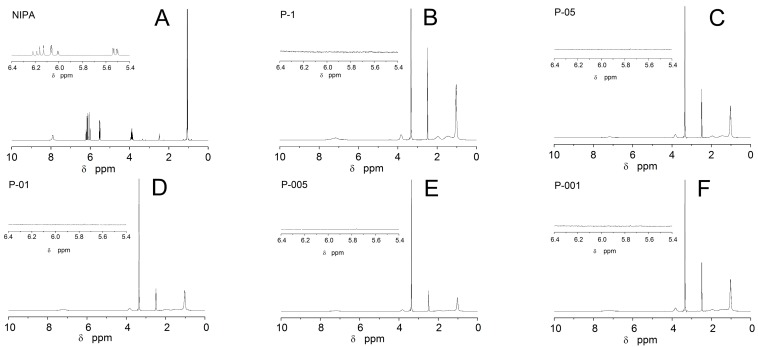
^1^H-NMR spectra of monomer NIPA and five synthesized polymers NIPA-(**A**); P1-(**B**); P05-(**C**); P01-(**D**); P005-(**E**); P001-(**F**). The expanded areas in the ^1^H-NMR spectra show the resonance range of the vinyl protons. δ: chemical shift.

Nuclear Magnetic Resonance Spectroscopy Measurements (^1^H-NMR) spectra of the sample NIPA monomer contain characteristic peaks at chemical shift δ 5.45–6.25 ppm, and originate from the protons of the unsaturated –C=CH groups. These signals are no longer visible on the spectrum of P-1, P-05, P-01, P-005, and P-001 polymers (cf. Figure 2B–F). The expanded vinyl regions of the ^1^H-NMR spectra were featured as inserts in the Figure 2A–F. In the spectrum of monomer NIPA, there were also signals of other protons at 1.00–1.10 ppm due to –CH_3_ groups, at 3.80–4.00 ppm from –CH– groups, and at 7.85–7.95 ppm assigned to the –NH. As expected, ^1^H-NMR spectra of synthesized polymers were similar in the terms of number and placement of the respective shifts characteristic for the protons reflecting the presence of functional groups. The observed variability of signal integration may reflect the amount of protons, which result in the recorded signal in a range 1–2 ppm. ^1^H-NMR (DMSO): for P-1, P-05, P-01, P-005, P-001, δ (ppm) 7.18 (–NH), 3.82–3.35 (–CH–), and 1.03 (–CH_3_).

### 2.4. Conductivity Measurements

**Figure 3:**
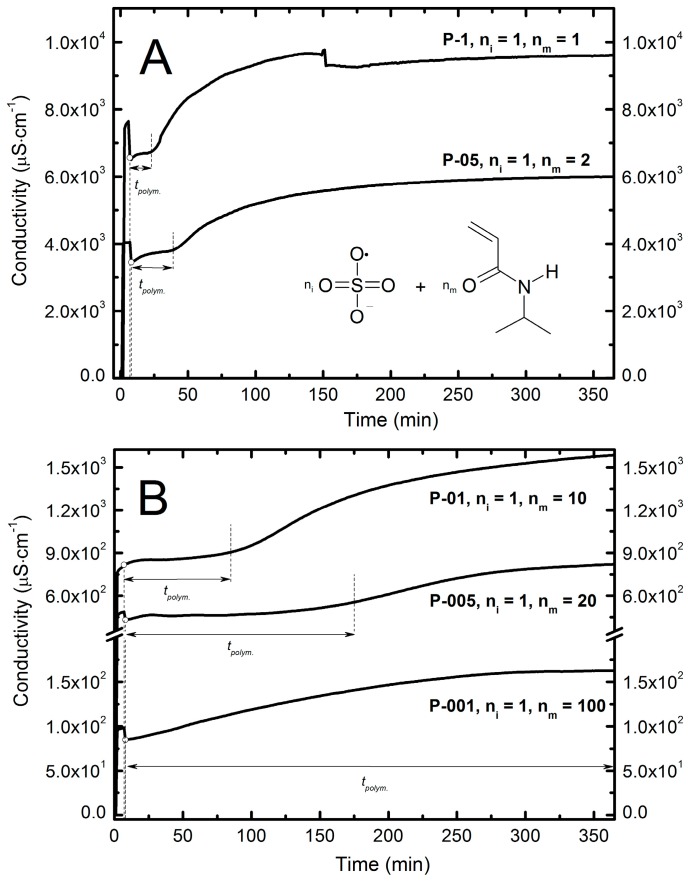
Dependence of conductivity in function of time during the entire period of polymerization reaction of P-1, P-05 (**A**) and P-01, P-005, P-001 (**B**) reaction mixtures. The symbols n_i_ and n_m_ represent the numbers of particles attributed respectively to the amount of initiator and to the amount of monomer, whereas the t_polym_ may reflect the initial formation of oligomeric radicals.

The progress of each polymerization reaction was monitored by measuring of conductivity of reaction mixture in the course of the synthesis. Figure 3A,B presents changes of conductivity of polymers P-1, P-05, P-01, P-005, and P-001 assessed in aqueous dispersions as a function of time.

Based on the graphic interpretation of results, some phases of the polymerization process may be reflected by the conductivity values. It was observed that the change in concentration of initiator strongly affects the length of observed stages of the polymerization. The chain initiation process, and the initial formation of oligomeric radicals were particularly influenced by the initiator concentration, and may be described as t_polym_ on the Figure 3A,B.

In four of the studied reaction systems—P-1, P-05, P-01, and P-005—the distinct turbidity was observed, whereas during the SFPP of P-001 we did not observe any turbidity. The low concentrations of the initiator resulted in a longer time between reaction onset and the appearance of the turbidity. In the reaction system P-1 turbidity occurred almost immediately after injecting the monomer solution, while in mixtures P-05, P-01, P-005 turbidity appeared respectively after ca. 4, 10, and 17 min. Conductivity values registered at the moment of the appearance of turbidity were 3540, 845, 465 μS·cm^−1^ for P-05, P-01, and P-005 respectively. As expected, the data of Figure 3A,B demonstrate that with increasing concentration of initiator increased the initial values of the conductivity and were 7420, 4020, 761, 458, 94.4 μS·cm^−1^, respectively for the P-1, P-05, P-01, P-005 and P-001. In each case, addition of monomer resulted in a small temporary decrease of conductivity followed by a noticeable increase. Time to the onset of plateau phase was different for each system.

According to the conductivity vs. time curves on Figure 3A,B it can be noted that for four systems—P-1, P-05, P-01, P-005—changes in conductivity proceeded stepwise and for P-1 and P-05 changes occurred abruptly, whereas in the cases of P-005 and P-001, they were rather prolonged. Conductivity in the P-001 system during the polymerization process increased linearly to achieve the constant value.

Values of conductivity of the P-1 system varied in a range from 6500 to 9700 μS·cm^−1^. The initiation process continued about 22 min and finished at a conductivity value of 6740 μS·cm^−1^, the next stage lasted 128 min, and the conductivity clearly increased to a constant value equal to 9700 μS·cm^−1^.

The P-05 conductivity in the course of the synthesis changed in the range of 3450 to 6000 μS·cm^−1^. A significant increase followed after 33 min from the start of the reaction, and the conductivity approached 3850 μS·cm^−1^. Constant value equal to 6000 μS·cm^−1^ appeared after 318 min.

The measured conductivity values for the P-01 system varied in the range from 761 to 6000 μS·cm^−1^. In these cases, we did not register establishment of a constant value of conductivity in the final stage of the reaction. The conductivity increased continuously until the end of synthesis. At the beginning of the process of polymerization, after addition of the monomer to the system, there was a rapid increase of conductivity lasting up to 10 min observed, up to value of 850 μS·cm^−1^. Then the value of conductivity was fixed for 20 min at 852 μS·cm^−1^ with slight deviations of ±1 μS·cm^−1^. The conductivity increased over 20 min to value of 901 μS·cm^−1^ and proceeded sharply over 83 min to a value of 1289 μS·cm^−1^ and then increased further continuously and gently to the end of synthesis achieving a value 6000 μS·cm^−1^.

The shape of the curve illustrating the changes of conductivity during the polymerization reaction for the system P-005 was similar to the shape of the analogous graph plotted for the P-01. However, there were clear differences in the duration of the individual polymerization stages. For the P-005 system, each step takes much longer. The conductivity, after addition of the monomer to the reaction vessel, decreased from 487 to 431 μS·cm^−1^ and then significantly increased to a value of 466 μS·cm^−1^. The increase stage lasts 18 min and is 8 min longer compared to the P-01 system. The intermediate state of 62 min has a constant value of conductivity equal to 466 μS·cm^−1^ (±4 μS·cm^−1^), similarly to the case of P-01. In the next 100 min, the conductivity increased slightly up to 530 μS·cm^−1^, followed by rapid increase in the next 110 min until 756 μS·cm^−1^. Above this value, the increment of conductivity change was slower but constantly increased until the end of the synthesis to achieve 821 μS·cm^−1^.

During the polymerization process, in the P-001 system, the conductivity increased slightly in the range 85–163 μS·cm^−1^, for 5 h and 36 min from the onset of the reaction.

### 2.5. Hydrodynamic Diameter and Polydispersity Index

The hydrodynamic diameter of resulting polymers—P-1, P-05, P-01, P-005, and P-001—was approximately 20, 22, 50, 63 and 530 nm, respectively.

**Figure 4:**
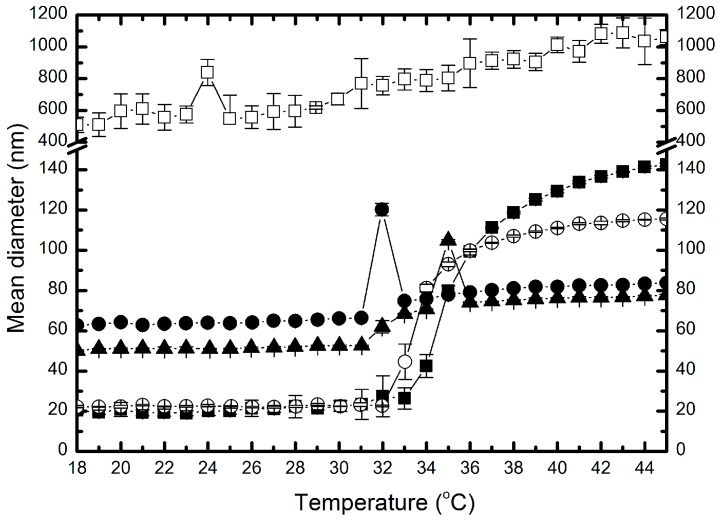
Hydrodynamic diameters of particles: P-1 (■); P-05 (○); P-01 (▲); P-005 (●) and P-001 (□) as a function of temperature.

Figure 4 presents common data of variability of hydrodynamic diameters of synthesized nanoparticles in aqueous suspension independent of temperature. Due to the data in Figure 4, hydrodynamic diameters of P-1 particle (■) increased from ca. 20 nm to 140 nm with the increasing temperature. However, between 18 and 32 °C the particle size was constant, equal to ca. 20 nm, with a fluctuation of ±3 nm. In the temperature range of 32–37 °C there was a sharp increase in particle size in the range of 27–111 nm observed. Over the temperature of 37 °C, hydrodynamic diameter increased slower and achieved the value of ca. 140 nm at 45 °C. In the case of polymer P-05 (○), the data of hydrodynamic diameter vs. temperature form a similar shapes as polymer P-1 (cf. Figure 4). In the range 18–45 °C the size of particles increased from 22 to 116 nm. Around temperature of 18 °C through the next 14 °C, the hydrodynamic diameter did not change distinctively and is equal to ca. 22 nm. However, the hydrodynamic diameter of the polymer P-05 increased sharply to 93 nm at 35 °C and then slightly to 115 nm at 45 °C. The hydrodynamic diameter of particles P-01 (▲) increased in a range of 50–78 nm, and one noticeable growth was around 62–104 nm in the temperature range 32–35 °C, whereas over 35 °C to 45 °C particles maintained a size 76 ± 1 nm.

Mean diameter-temperature curve (●) for nanoparticles P-005 is similar to polymer P-01. The particles size increased from 62 to 84 nm in the range 18–45 °C, however the intensive growth of hydrodynamic diameter actually occurs in the temperature range 31–35°C. Above the temperature of 35 °C, the particles achieved a size of 81 nm, very close to size of P-01, in the same temperature. For P-001 (□), in the entire temperature range of 18–45 °C the hydrodynamic diameter tended to increase from 450 to 1100 nm without any pronounced and characteristic leap.

**Figure 5:**
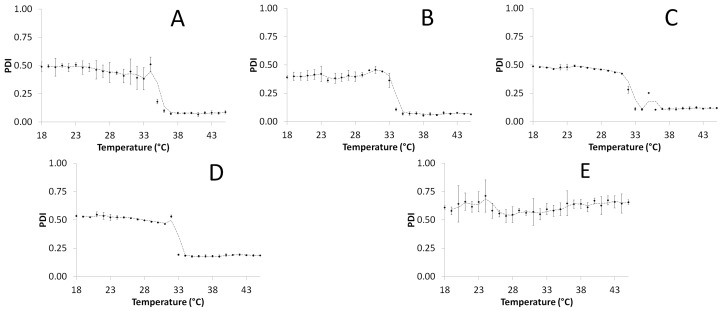
The effect of the temperature on the polydispersity index (PDI) of the synthesized nanospheres P1-(**A**); P05-(**B**); P01-(**C**); P005-(**D**); P001-(**E**) obtained from dynamic light scattering (DLS) measurements.

The studied polymer particles additionally were characterized by determination of polydispersity index, which was in the range 0.39–0.61 at 18 °C. In the case of polymers P-1, P-05, P-01, and P-005, the polydispersity index decreased with increasing temperature with a significant drop around 33 °C (cf. Figure 5A–D). The polydispersity of the P-001 remained at the constant level ca. 0.61.

### 2.6. Zeta Potential

Zeta potential (ZP) of all synthesized polymers was measured as a function of temperature in the range of 18–45 °C in aqueous solution.

**Figure 6:**
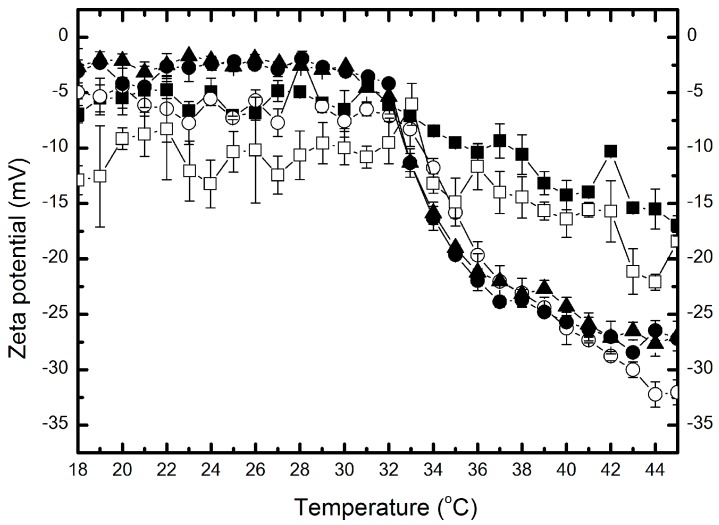
The effect of temperature on the zeta potential of P-1 (■); P-05 (○); P-01 (▲); P-005 (●) and P-001 (□) polymers.

The values of ZP of P-1, P-05, P-01, P-005, and P-001 particles were negative in the whole measuring temperature range, and at 18 °C they were found to be around: −6.94, −4.97, −2.69, −3.08, and −12.9 mV, respectively. Figure 6 presents characteristic trends showing temperature dependence of ZP for the assessed polymers. Between 18 °C and 32 °C the value of ZP remained stable with minimal variations. Further, with an increase of temperature, the values of ZP decreased to values of −17, −32, −30, −27 and −18 mV, for P-1, P-05, P-01, P-005 and P-001 polymers, respectively. Variations (∆ZP) between initial and final values of ZP for samples P-05, P-01 and P-005 are close each to other, and definitely higher compared to P-1 and P-001. The results are gathered in Table 1.

Experimental data points depicted in the Figure 6 reveal that the temperature 32 °C is the same for all samples in that the values of ZP decreased sharply. This indicates that nanoparticles started to undergo the volume phase transition.

## 3. Discussion

According to the performed FTIR studies, the vinyl bonds were saturated during the performed SFPP procedure. The course of polymerization reaction was additionally proven by ^1^H-NMR assessment. As shown in this work, the conductivity measured during the polymerization process may be used to distinguish some of its stages. The initial decrease in conductivity after the addition of the monomer may appear due to the formation of macroradicals. According to a wide bibliography, the stage of macroradical formation takes place quickly and covers the attachment of radicals derived from the initiator molecules with monomer particles. Numerous studies confirmed that rate of this process *V* is proportional to the square root of the initiator concentration [*I*] [24], as well as to the concentration of monomer [*M*], respective to Equation (1):

(1) $$V=k\cdot \left[M\right]\cdot {\left[I\right]}^{0.5}$$

In order to determine the possibilities of using the results of conductivity measurements to assess the course of the reaction SFPP, we determined the relationship between the observed rate of the process (Figure 3A,B) measured by conductometry, and the square root of the concentration of initiator ([*I*]^0.5^) (Table 2).

**Figure 7:**
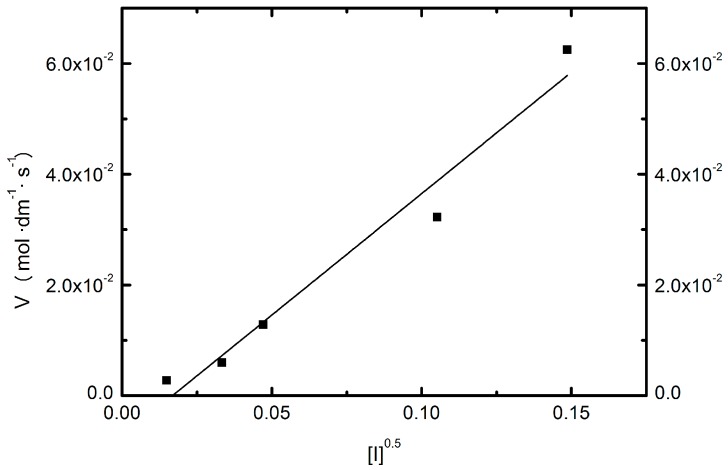
Relationship of the reaction rate of polymerization NIPA (*V*) and [*I*]^0.5^.

As it is apparent from the obtained relation (Figure 7), the rate of this reaction step was proportional to the square root of the concentration of the initiator, which was confirmed by the high value of the square of the Pearson coefficient, amounting to 0.9570. This relationship is expressed by Equation (2):

*y* = 0.4385*x* − 0.0074 (2)

**Figure 8:**
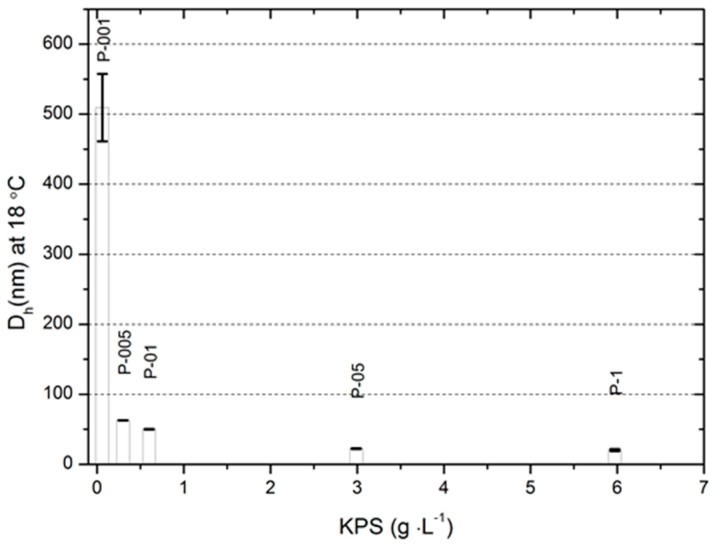
Particles size of prepared NIPA polymers P-1, P-05, P-01, P-005 and P-001 as a function of concentration of initiator potassium persulphate (KPS).

According to the theory of free radical polymerization, it can be assumed that in the studied process, the first stage (Figure 3A,B) corresponds to the phase of attachment of the radical to monomer molecules. This is confirmed by systematically increasing the duration of the process, together with decreasing concentration of the initiator. Also, a sharp increase in conductivity, after the completion of the first phase, shows that molecules of initiator unused in the SFPP process undergo hydrolysis reaction. Ions formed in this stage resulted in high conductivity in all tested systems with the exception of P-001. Figure 8 presents the influence of the initiator concentration on the particle size. The particles prepared at higher concentrations of KPS feature a low average hydrodynamic diameter. At higher concentrations of initiator, the obtained hydrodynamic diameter was equal to ca. 20 nm, whereas in the case of lowest amount of initiator the size ranged ca. 500 nm.

These results indicate that size of poly-NIPA particles can be adjusted by the concentration of the initiator, and indirectly conforms, that the conductivity measurements well correspond with the resulting diameters of synthesized NIPA derivatives. The decreasing PDI observed in the course of heating of the polymeric particles, related with respective decrease of absolute ZP indicates enhanced stability of the polymeric particles at temperatures exceeding the VPTT. According to the data on the Figure 4 the VPTT was in the range reflecting the thermal conditions on the human skin surface [25], and the initiator concentration did not influence the value of VPTT. Obtained data may enrich the databases for further research in the field of kinetic modelling, which was presented by other authors [26].

## 4. Materials and Methods

### 4.1. Materials

*N*-isopropylacrylamide (NIPA, 99%) was purchased from Sigma-Aldrich Chemical (St. Louis, MO, USA). Potassium persulfate (KPS, 99%) was supplied from Sigma-Aldrich Chemical (Tokyo, Japan). All chemicals were used as received. Dialysis membrane with a pore size of molecular weight cut-off (MWCO): 12,000–14,000 Da was obtained from Sigma-Aldrich Chemical (St. Louis, MO, USA). Deionized water was obtained from a 0.22 μm microfiltration capsule in a HLP 20 device from Hydrolab (Straszyn, Poland), fulfilling requirements of PN-EN ISO 3696:1999 for analytical laboratories.

### 4.2. Synthesis of Poly-N-isopropylacrylamide Particles

The poly-*N*-isopropylacrylamide (polyNIPA) particles were prepared in an aqueous solution using KPS as a redox initiator. Increasing amounts of powdered KPS (cf. Table 3) were dissolved in 900 mL of distilled water heated up to 70 °C in a 2000-mL four-necked round bottom flask equipped with magnetic paddle stirrer, reflux condenser, thermometer, conductivity cell (*K* = 1 cm^−1^) and nitrogen gas inlet. The solution of initiator was stirred (250 rpm) and degassed with N_2_ for ca. 10 min. Next, monomer NIPA (ca. 5.000 g) was dissolved in 100 mL of distilled water and added continuously to the formerly prepared solution of initiator. The reaction vessel was kept at 70 °C for 6 h with respective mixing. After the reaction was terminated, the mixture was cooled to room temperature. Obtained polymer solutions were purified via dialysis for 15 days at room temperature. The water was stirred and changed every 24 h. The purified products were frozen and freeze-dried by Alpha 1–2 LD (Martin Christ Freeze Dryers, Osterode am Harz, Germany) for 32 h.

### 4.3. Fourier Transform Infrared Spectroscopy

FTIR spectra of the dry polymers were recorded using a Fourier transformed infrared spectroscopy with attenuated total reflectance (ATR-FTIR) technique (Nicolet iS50 FT-IR spectrometer, Thermo Fisher Scientific Madison, WI, USA) equipped with deuterated L-alanine doped triglycine sulfate with KBr window in the range of 400 cm^−1^ to 4000 cm^−1^ at 32 scans per sample and a resolution of 4 cm^−1^. Background readings were collected and subtracted from each spectrum before data output.

### 4.4. Nuclear Magnetic Resonance Spectroscopy

The ^1^H-NMR spectra of the polymer were recorded using a Spectrometer Bruker 300 MHz (Bruker, Rheinstetten, Germany). The solutions of polymers and monomer were prepared by dissolving about 10 mg of each compound in 7 mL of DMSO-d6 (δ = 2.49). Temperature of the analysis was 26 °C.

### 4.5. Conductivity Measurements

The conductivity of reaction mixture was measured in the course of the reaction by using a conductivity cell (*K* = 1.00 cm^−1^) connected to a laboratory conductivity meter model CC-505 (Elmetron, Zabrze, Poland). Measurements were performed at 70 °C with meter accuracy of 0.1% and, for values bigger than 20 mS·cm^−1^, accuracy was 0.25%. The conductivity cell for the whole reaction was in contact with reaction solution. Conductivity was measured as a function of time and recorded on PC.

### 4.6. Dynamic Light Scattering

Dynamic Light Scattering (DLS) Zeta Sizer Nano device of Malvern Instruments was used to measure both values of hydrodynamic diameter and ZP in water dispersions of the synthesized polymers as a function of temperature. Hydrodynamic diameter was recorded using dynamic light scattering as a basic technique and ZP was measured using Doppler electrophoresis method. The laser was used at a wavelength of λ = 678 nm, and the light angle was 90°. Measurements were carried out over the temperature range of 18–45 °C, and at each temperature the samples were equilibrated for 120 s. For the size measurements, a DTS-0012 cuvette (Malvern Instruments, Malvern, UK) was filled by 1 mL of sample of the derived polymer concentration. ZP was measured in capillary cell type DTS-1070 (Malvern Instruments, Malvern, UK). Values of particle size were expressed as the mean of five measurements and data of ZP were averaged from three replicates. The polydispersity index of the synthesized NIPA polymers was excavated from the dynamic light scattering measurements using ZetasizerNano Software, Version 7.11 (Malvern Instruments, Malvern, UK).

## 5. Conclusions

Due to applied FTIR, NMR, and DLS assays in the course of polyNIPA SFPP with the use of anionic initiator KPS in the range of 2.21 × 10^−2^–2.22 × 10^−4^ mol·L^−1^, the polymeric nanoparticles are formed. The course of free radical polymerization can be followed using conductivity measurements. Initiator concentration affects the size and ZP of particles, and this fact is reflected in the observed values of conductivity in the course of the reaction. The changes in ZP values reflect the volume phase transition and may be applied for the determination of VPTT. At increased temperatures overcoming VPTT, the obtained particles have enhanced stability due to the PDI and EKP results. The obtained series of data will be applied for further development of thermosensitive nanocariers for thermally triggered drug delivery.

## Figures and Tables

**Table 1:** Zeta potential (ZP) and the differences between values of zeta potential (∆ZP) at the initial and final temperature of measurement.

Type of Polymer	Range of ZP (mV)	∆ZP (mV)
P-1	−7 to −17	10
P-05	−5 to −32	27
P-01	−3 to −30	27
P-005	−3 to −27	24
P-001	−12 to −18	6

**Table 2:** Evaluated time of radical polymerization for polymers P-1, P-05, P-01, P-005, and P-001 and the square root of the initiator concentration ([*I*]^0.5^) used in synthesis.

Type of Polimer	Estimated Time of Polymerization (min)	${\left[I\right]}^{0.5}$
P-1	16	0.1486
P-05	31	0.1052
P-01	78	0.0471
P-005	167	0.0333
P-001	363	0.0149

**Table 3:** Substrate content of prepared nanospheres: P-1, P-05, P-01, P-005, and P-001.

Type of Nanosphere	NIPA (g)	KPS (g)
P-1	5.002	5.972
P-05	5.004	2.986
P-01	5.006	0.598
P-005	5.002	0.300
P-001	5.006	0.061
